# Brugada Syndrome-Associated Genetic Loci Are Associated With J-Point Elevation and an Increased Risk of Cardiac Arrest

**DOI:** 10.3389/fphys.2018.00894

**Published:** 2018-07-10

**Authors:** Laura Andreasen, Jonas Ghouse, Morten W. Skov, Christian T. Have, Gustav Ahlberg, Peter V. Rasmussen, Allan Linneberg, Oluf Pedersen, Pyotr G. Platonov, Stig Haunsø, Jesper H. Svendsen, Torben Hansen, Jørgen K. Kanters, Morten S. Olesen

**Affiliations:** ^1^Danish National Research Foundation Centre for Cardiac Arrhythmia, Copenhagen, Denmark; ^2^Laboratory for Molecular Cardiology, Department of Cardiology, The Heart Centre, Rigshospitalet, Copenhagen University Hospital, Copenhagen, Denmark; ^3^Faculty of Health and Medical Sciences, Novo Nordisk Foundation Center for Basic Metabolic Research, University of Copenhagen, Copenhagen, Denmark; ^4^Research Centre for Prevention and Health, Copenhagen, Denmark; ^5^Department of Clinical Experimental Research, Rigshospitalet, Glostrup, Denmark; ^6^Department of Clinical Medicine, Faculty of Health and Medical Sciences, University of Copenhagen, Copenhagen, Denmark; ^7^Center for Integrative Electrocardiology at Lund University, Arrhythmia Clinic, Skåne University Hospital, Lund, Sweden; ^8^Department of Medicine and Surgery, Faculty of Health Sciences, University of Copenhagen, Copenhagen, Denmark; ^9^Laboratory of Experimental Cardiology, Department of Biomedical Sciences, University of Copenhagen, Copenhagen, Denmark

**Keywords:** Brugada Syndrome, single nucleotide polymorphism, electrocardiogram, general population, mortality

## Abstract

**Introduction:** A previous genome-wide association study found three genetic loci, rs9388451, rs10428132, and rs11708996, to increase the risk of Brugada Syndrome (BrS). Since the effect of these loci in the general population is unknown, we aimed to investigate the effect on electrocardiogram (ECG) parameters and outcomes in the general population.

**Materials and Methods:** A cohort of 6,161 individuals (median age 45 [interquartile range (IQR) 40–50] years, 49% males), with available digital ECGs, was genotyped and subsequently followed for a median period of 13 [IQR 12.6–13.4] years. Data on outcomes were collected from Danish administrative healthcare registries. Furthermore, ~400,000 persons from UK Biobank were investigated for associations between the three loci and cardiac arrest/ventricular fibrillation (VF).

**Results:** Homozygote carriers of the C allele in rs6800541 intronic to *SCN10A* had a significantly larger J-point elevation (JPE) compared with wildtype carriers (11 vs. 6 μV, *P* < 0.001). There was an additive effect of carrying multiple BrS-associated risk alleles with an increased JPE in lead V1. None of the BrS-associated genetic loci predisposed to syncope, atrial fibrillation, or total mortality in the general Danish population. The rs9388451 genetic locus adjacent to the *HEY2* gene was associated with cardiac arrest/VF in an analysis using the UK Biobank study (odds ratio = 1.13 (95% confidence interval: 1.08–1.18), *P* = 0.006).

**Conclusions:** BrS-associated risk alleles increase the JPE in lead V1 in an additive manner, but was not associated with increased mortality or syncope in the general population of Denmark. However, the *HEY2* risk allele increased the risk of cardiac arrest/VF in the larger population study of UK Biobank indicating an important role of this common genetic locus.

## Introduction

Recent advances in the field of genetics have led to an increased focus on the genetic component of cardiac diseases associated with sudden cardiac death (SCD) (Ghouse et al., 2015; Manrai et al., 2016). Genetic testing has become an important tool in the clinical setting, contributing to the identification of patients and family members at risk of having an inherited cardiac disease. Brugada Syndrome (BrS) is an inherited arrhythmic disease characterized by ST-segment elevations in leads V1-V2 and an increased risk of ventricular arrhythmias and sudden cardiac death (Nielsen et al., 2013). Currently more than 20 genes have been associated with BrS, although very few monogenic forms of BrS have been identified (Watanabe and Minamino, 2016).

A recent genome-wide association study (GWAS) identified three loci (rs9388451 in proximity to *HEY2*; T>C, rs10428132 close to *SCN10A*; T>G, and rs11708996 close to *SCN5A*; G>C) to be associated with BrS. Patients carrying more than four of these risk alleles had an odds ratio (OR) of 21.5 for BrS, compared with patients carrying zero or one risk allele. All three GWAS-identified loci are presumed to affect the sodium current and, in addition, the *HEY2* loci has been associated with diminished transmural differences in the *I*_to_ current in cardiac myocytes (Bezzina et al., 2013; Veerman et al., 2017). Since these three variants are common also in the background population, our aim was to investigate the effect of these single nucleotide polymorphisms (SNPs) on the electrocardiogram (ECG), syncope, BrS, and/or atrial fibrillation (AF), and mortality in the general population.

## Material and methods

### Study population Inter99

The study population was drawn from the Inter99 study. The Inter99 study was a population-based randomized lifestyle intervention trial (CT00289237, ClinicalTrials.gov) investigating the effects of lifestyle intervention on the risk of cardiovascular death. Genotyping was performed on 6,161 individuals from the Inter99 study cohort (Glümer et al., 2003). All participants have given their informed consent and were, by self-report, of Danish origin. The study population included individuals above the age of 29 years. The study design has previously been described in detail (Jørgensen et al., 2003). The protocol was in accordance with the Helsinki declaration and approved by the local ethical committee.

### Study population UK biobank

The three genetic loci were further investigated in individuals from the open resource UK Biobank study including 408,961 white British participants with European ancestry, recruited between 2006 and 2010. The UK Biobank project is a large prospective cohort study of approximately 500,000 individuals from the United Kingdom, aged between 40 and 69 at recruitment. Additional to blood, saliva, and urine samples, the study contains self-reported information including diet and exercise habits, physical and cognitive measurements, and data from medical records and registries (Sudlow et al., 2015).

### Genotyping Inter99

The rs11708996 SNP was genotyped on the Illumina Human Exome Chip 12 v1.0. using the Genotyping module (version 1.9.4) of GenomeStudio software (version 2011.1, Illumina) and custom cluster data generated from 17,621 Danish DNA samples analyzed on the same Illumina HiScan. Since the rs10428132 SNP was not available on the Exome Chip, another SNP in complete linkage (rs6800541, 1.0 *r*^2^) was chosen, and the abovementioned genotyping was performed. Individuals were excluded during quality control (QC) by removing closely related individuals, individuals with an extreme inbreeding coefficient, individuals with a low call-rate <90%, individuals with a mislabeled sex and individuals with a high discordance rate to previously performed genotypings. Both SNPs were of high quality and in Hardy-Weinberg equilibrium (HWE) *P* > 0.05 and call-rates above 99.9%. European ancestry was confirmed using principal component analysis (PCA, Supplementary Figure 1). The rs9388451 SNP was not available on the Human Exome Chip and the same individuals who passed the abovementioned QC was therefore genotyped using KASP TM bi-allelic discrimination (LGC Genomics, Herts, UK), resulting in high quality genotypes (call-rate 99.3%, HWE *P* = 0.13).

### Genotyping UK biobank

Genotyping was performed using Applied Biosystems UK BiLEVE Axiom Array by Affymetrix in the first 50,000 participants, as described previously (Wain et al., 2015), and using the closely related Affymetrix UK Biobank Axiom Array in the remaining participants (Welsh et al., 2017). Both arrays were custom-made and share 95% of marker content^1^. Several tests for marker-based and sample-based QC were performed in order to account for effects such as population structure and batch-based genotype calling, including tests for PCA, batch effects, plate effects, departures from HWE, sex effects, array effects, discordance across control replicates, extreme heterozygosity and high missing rates, and relatedness. Further details on QC have been described previously^1^. Imputation was performed by Wellcome Trust Centre for Human Genetics using reference panels from 1000Genomes phase 3 and the Haplotype Reference Consortium (HRC), as described previously^1^.

### ECG data analyses Inter99

All Inter99 participants underwent ECG recording upon inclusion and ECGs were recorded with the patient at rest and in a supine position. The V1 and V2 recordings were placed at the 4th intercostal space. All ECGs were digitally recorded and stored in the MUSE Cardiology Information System (GE Healthcare, Wauwatosa, Wisconsin) and later processed using version 21 of the Marquette 12SL algorithm.

ECG parameters were assessed digitally, including heart rate, PR-interval, QRS-duration, QTc-interval, and J-point elevation (JPE) and depression in lead V1 and V2. The QT-interval was corrected for heart rate using Bazett's formula (QTcB) (Sagie et al., 1992). Extreme outliers outside mean ± 5 standard deviations (SD) were removed (PR-interval; *n* = 6, QRS-duration; *n* = 17, QTc-interval; *n* = 2, JPE V1; *n* = 11, JPE V2; *n* = 8).

In addition, two experienced physicians analyzed all ECGs manually and independently for the diagnostic type 1 Brugada pattern (Priori et al., 2015).

### Syncope, arrhythmia, and all-cause mortality Inter99

All Danish residents are assigned a personal and unique civil registration number, which enables linkage to Danish nationwide registries on an individual level. The Danish National Patient Registry (NPR) contains information on all in- and out-of-hospital patient activities in Denmark since 1978. The civil registration number was used to access register data in NPR using the International Classification of Diseases, 10th revision (revised in 1994; ICD-10). We identified all Inter99 patients who prior to or during follow-up had a hospital, out-patient clinic, or emergency room discharge diagnosis with one of the following ICD-10 codes: syncope (DR559), AF (DI48), BrS (DI472M), and cardiac arrest (DI46). Information regarding all-cause mortality was extracted from the Danish Cause of Death Registry (Helweg-Larsen, 2011) containing data on all deaths among Danish citizens living in Denmark, Greenland, and the Faroe Islands since 1970. Patients were included from 1999 to 2001 and followed until December 17, 2012 when data were retrieved from Danish nationwide health care registries for assessment of the clinical outcome.

### Statistics Inter99

Continuous variables were assessed using ANalysis Of Variance (ANOVA) adjusted for age and sex. Follow-up time began on the day of inclusion and ended at death, emigration, or end of follow up (December 17, 2012), whichever came first.

For each SNP, Cox regression was used to assess the risk of syncope, AF, and death when carrying one or two risk alleles compared with carrying zero risk alleles, assuming an additive model. Since individuals were included from the age of 30, left-truncation was used. To assess the effect of the three SNPs, individuals were categorized into having 0, 1–2, 3–4, or 5–6 risk alleles and compared with having zero alleles with a Dunnett's Post Hoc Test.

The survival plots for each variant illustrate the probability of survival, when harboring a BrS-associated variant compared with not harboring a BrS-associated variant. The combined survival plot only includes individuals with succeeded genotyping of all three variants. Individuals with genotyping, but without available ECG, are included in the survival analyses. The two-tailed log-rank test was used to test the difference in overall mortality between the groups.

A *P*-value of less than 0.05 was considered significant. Since three SNPs were investigated, Bonferroni correction for multiple testing was applied. Hence a *P*-value of 0.05/3 = 0.017, was needed to obtain statistical significance. Statistical analyses were conducted using R, version 3.3.2.

### Analyses UK biobank

The three SNPs were further investigated using the tools in the UK Biobank Michigan PheWeb browser (PheWeb, 2018). The PheWeb browser has been generated using the UK Biobank Resource under application number 24460 and enables us to analyse the phenotypic associations between the three SNPs and cardiac arrest/VF in individuals from UK Biobank. The analyses were limited to the ethnic majority of UK Biobank consisting of 382,000 white individuals to avoid population stratification biases. To avoid large type I error rates in the analysis of phenotypes with imbalanced case-control ratios, a generalized mixed model association test, SAIGE, has been developed and applied to the UK Biobank dataset. The model also takes sample relatedness into account, and has previously been described in detail^2^.

## Results

### Inter99

Genotyping was done in 6,161 individuals, and was successful of all three SNPs in 6,068 individuals. Of these, 73 individuals had no ECG.

Baseline characteristics of the study population are shown in Table 1. The associations between the BrS-associated genetic loci and the JPE in V1 and V2, QTcB, PR-interval, and QRS-duration are presented in Table 2. Risk allele frequencies (RAFs) are 0.15 for rs11708996, 0.38 for rs6800541, and 0.49 for rs9388451, respectively.

**Table 1:** Baseline characteristics of the study population.

**Clinical characteristics**	
**PARTICIPANT INFORMATION**	
Number of subjects	6,161
Male gender, n (%)	3,020 (49)
Median age, years (IQR)	45 (40–50)
Median BMI (IQR)	26 (23–29)
**ECG MARKERS**	
Heart rate, bpm (mean ± SD)	67.2 ± 11.3
Sinus bradycardia, n (%)^†^	1,614 (27)
Brugada type 1 pattern, n^‡^	1 (0.02)

*BMI, body mass index; bpm, beats per minute; IQR, interquartile range; n, total number of individuals; SD, standard deviation*.

† *Sinus bradycardia defined as heart rate <60 bpm*.

‡ *Manually assessed*.

**Table 2:** Effect size of Brugada Syndrome-associated variants on various ECG markers.

**Nearest gene**	**Bp substitution, risk allele**	**rs#**	**Number of subjects with available ECGs**	**Alleles**	**Mean J-point elevation in lead V1**, μ**V**			**Mean J-point elevation in lead V2**, μ**V**			**Mean QTcB, ms**		
					**Mean, (n)**	**95% CI**	***P*-value**	**Mean, (n)**	**95% CI**	***P*-value**	**Mean, (n)**	**95% CI**	***P*-value**
*SCN5A*	G/C, C	rs11708996	6086	Allele level									
				0	7.2, (4,385)	(6.3–8.0)		38.5, (4,387)	(37.0–40.0)		424.1, (4,393)	(423.5–424.8)	
				1	8.2, (1,548)	(6.8–9.7)	0.15	39.8, (1,549)	(37.4–42.2)	0.18	422.2, (1,549)	(421.1–423.4)	0.001^†^
				2	10.8, (141)	(6.4–15.1)	0.13	43.3, (141)	(36.1–50.1)	0.17	418.9, (141)	(415.2–422.6)	0.003^†^
*SCN10A*	C/T, C	rs6800541	6087	Allele level									
				0	5.8, (2,353)	(4.6–7.0)		36.5, (2,353)	(34.5–38.5)		424.4, (2,353)	(423.5–425.1)	
				1	7.8, (2,801)	(6.7–8.8)	0.02^†^	39.1, (2,803)	(37.3–40.9)	0.1	423.1, (2,808)	(422.2–423.9)	0.06
				2	11.1, (921)	(9.2–13.0)	<0.001^†^	44.8, (922)	(41.5–48.0)	<0.001^†^	422.6, (923)	(421.1–424.1)	0.06
*HEY2*	T/C, C	rs9388451	5996	Allele level									
				0	6.9, (1,522)	(5.5–8.4)		38.8, (1,522)	(36.3–41.3)		424.0, (1,526)	(422.8–425.2)	
				1	8.1, (3,052)	(7.0–9.1)	0.25	39.5, (3,052)	(37.7–41.3)	0.89	423.3, (3,054)	(422.5–424.1)	0.49
				2	6.9, (1,411)	(5.4–8.4)	0.87	37.8, (1,413)	(35.3–40.4)	0.44	423.3, (1,413)	(422.1–424.4)	0.42
**Nearest gene**	**Bp substitution, risk allele**	**rs#**	**Number of subjects with available ECGs**	**Alleles**	**Mean PR-interval, ms**			**Mean QRS duration, ms**					
					**Mean, (n)**	**95% CI**	***P*****-value**	**Mean**	**95 % CI**	***P*****-value**			
*SCN5A*	G/C, C	rs11708996	6086	Allele level									
				0	157.1, (4,372)	(156.4–157.7)		91.4, (4,380)	(91.0–91.7)				
				1	160.0. (1.541)	(158.9–161.2)	<0.001^†^	92.6, (1,547)	(92.1–93.2)	<0.001^†^			
				2	160.2, (138)	(156.8–163.5)	0.08	92.2, (141)	(90.6–93.9)	0.22			
*SCN10A*	C/T, C	rs6800541	6087	Allele level									
				0	155.3, (2,337)	(154.4–156.1)		90.9, (2,352)	(90.5–91.3)				
				1	158.7, (2,795)	(157.9–159.5)	<0.001^†^	92.1, (2,798)	(91.7–92.5)	<0.001^†^			
				2	162.3, (920)	(160.8–163.7)	<0.001^†^	92.6, (919)	(91.9–93.3)	<0.001^†^			
*HEY2*	T/C, C	rs9388451	5996	Allele level									
				0	157.0, (1,520)	(155.9–158.1)		92.0, (1,520)	(91.4–92.5)				
				1	157.9, (3,039)	(157.1–158.7)	0.24	91.8, (3,049)	(91.4–92.1)	0.30			
				2	158.9, (1,403)	(157.7–160.1)	0.03^†^	91.2, (1,410)	(90.7–91.8)	0.02^†^			

*Outliers outside mean ± 5 SD were not included in the calculations (PR-interval; n = 6, QRS-duration; n = 17, QTc-interval; n = 2, JPE V1; n = 11, JPE V2; n = 8). bp, basepair; CI, confidence interval; ECG, electrocardiogram; QTcB, QT interval corrected with Bazett's formula*.

† *statistically significant, P-value < 0.05. Age and sex adjusted*.

Distribution plots of the JPE in V1 and V2, PR-interval, and QRS-duration are shown in Supplementary Figure 2.

### Registry data on syncope, AF, BrS, and all-cause mortality

The median follow-up time was 13 years (interquartile range (IQR) 12.6–13.4 years). The number of registered events of syncope, AF, BrS, and cardiac arrest in the study population is shown in Table 3.

**Table 3:** Number of registered events in Danish health care registries, (*n* = 6,161).

**Event**	***N***
AF	144
BrS	0
Cardiac arrest	19
Syncope	144
Death from all causes	286

*AF, atrial fibrillation; BrS, Brugada Syndrome*.

There was no significant effect of carrying BrS risk alleles on the risk of AF [hazard ratio (HR) 0.94 per allele (CI 0.81–1.09)] or syncope [HR 1.02 per allele (CI 0.88–1.18)].

**Figure 1:**
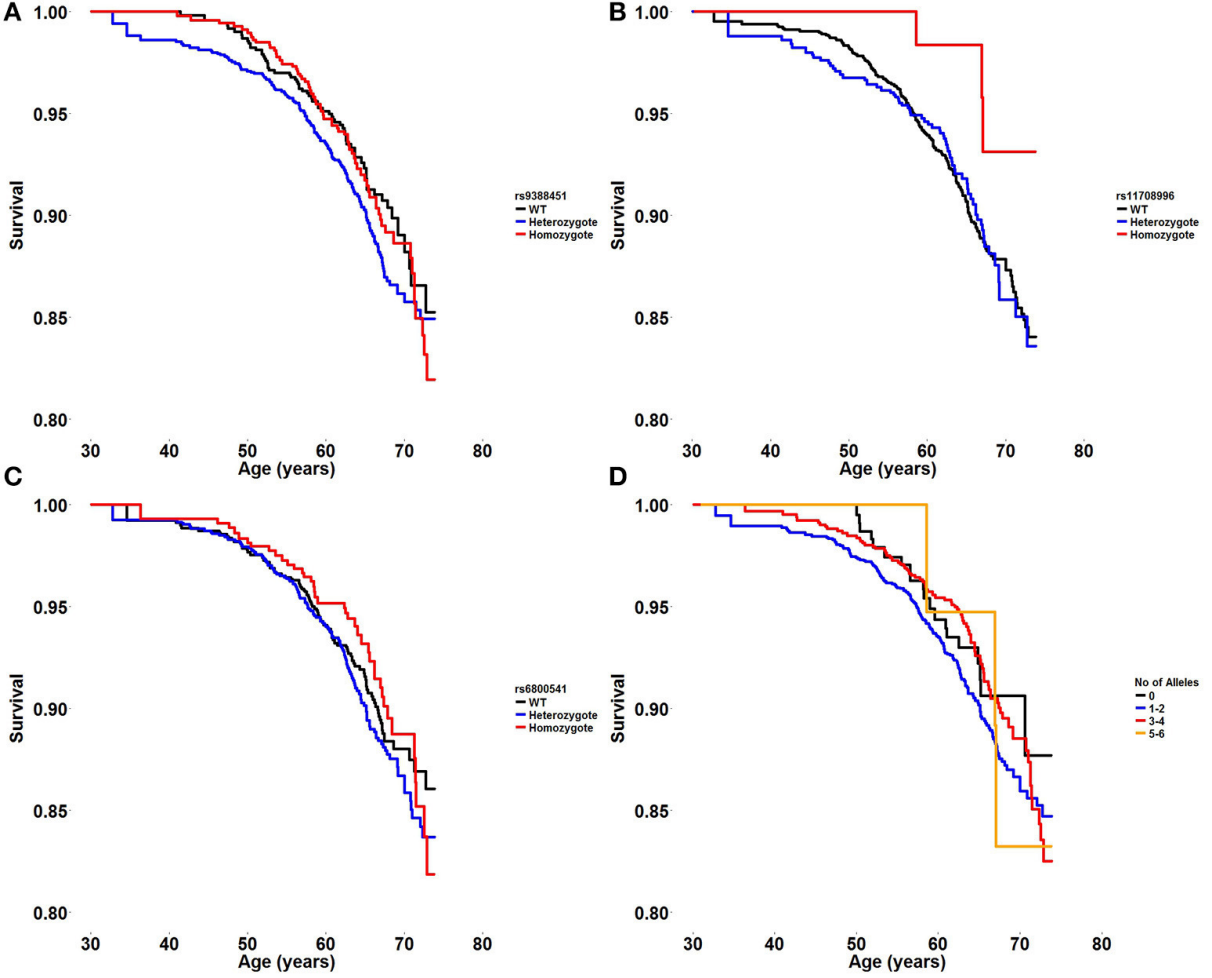
**(A–C)** Survival plot displaying the probability of survival during follow-up in different age groups in wildtype, heterozygote, and homozygote carriers of three BrS risk alleles; *HEY2* rs9388451, *SCN5A* rs11708996, and *SCN10A* rs6800541. **(D)** Survival plot displaying the probability of survival during follow-up in different age groups when carrying 0, 1–2, 3–4, or 5–6 BrS risk alleles.

Survival plots displaying the probability of survival during follow-up in different age groups are shown in Figure 1. There was no significant association between each risk variant and the hazard risk of death from all causes [rs6800541; HR 1.01 (95% CI 0.86–1.19), *P* = 0.9, rs9388451; HR 1.04 (95% CI 0.88–1.23), *P* = 0.6, rs11708996; HR 0.83 (95% CI 0.65–1.06), *P* = 0.14]. Furthermore, examining the additive effect of carrying multiple BrS-associated risk alleles, there was no difference in mortality when carrying 0, 1–2, 3–4, and 5–6 alleles [HR 0.95 per allele category, (95% CI 0.78–1.15), *P* = 0.57].

### Effect on J-point elevation

Homozygote carriers of the *SCN10A* rs6800541 variant had an increase in JPE compared to wildtype in both lead V1 and V2 (5 μV, 95% CI, *P* < 0.001 and 8 μV, *P* < 0.001, respectively) (Table 2). Heterozygote carriers of rs6800541 were not associated with an increased JPE in lead V2, but had a borderline significant JPE in lead V1 compared to non-carriers. Harboring any of the *SCN5A* and *HEY2* variants did not significantly affect the J-point.

**Figure 2:**
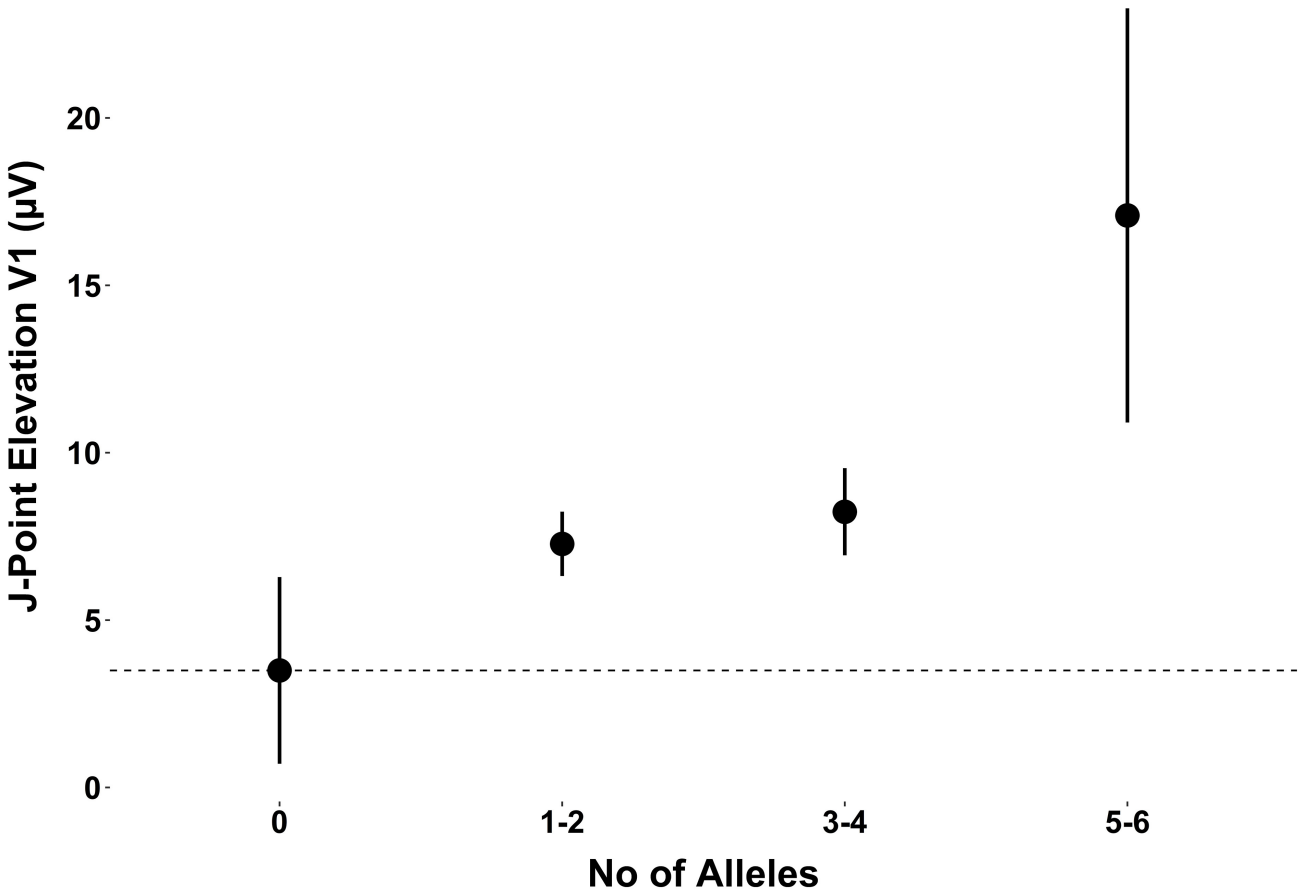
The additive effect of carrying multiple BrS-associated risk alleles on the JPE in lead V1. JPE (mean ± SE) as a function of number of risk alleles (*SNC5A* rs11708996, *SCN10A* rs6800541, *HEY2* rs9388451). JPE, J-point elevation; SNP, single nucleotide polymorphism.

**Figure 3:**
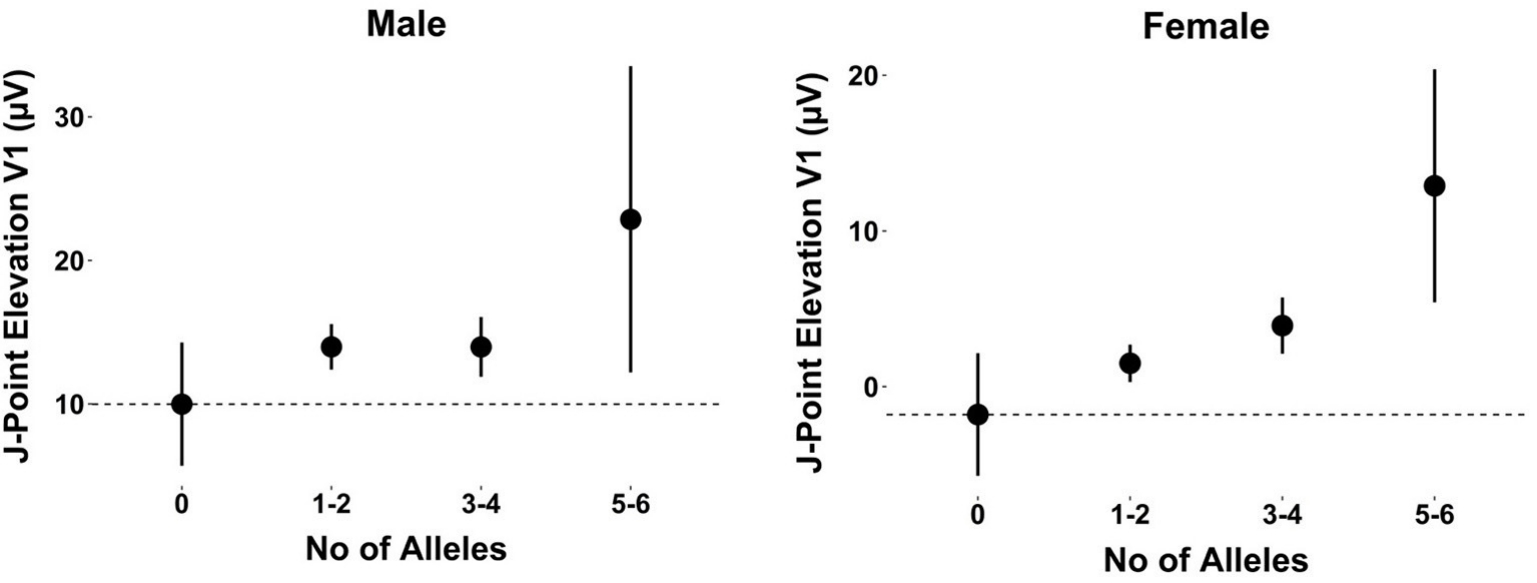
The additive effect of carrying multiple BrS-associated risk alleles on the JPE in lead V1 for males and females. JPE (mean ± SE) as a function of number of risk alleles (*SNC5A* rs11708996, *SCN10A* rs6800541, *HEY2* rs9388451). JPE, J-point elevation; SNP, single nucleotide polymorphism.

When observing the additive effect of carrying multiple BrS-associated risk alleles, there was a dose-response association, with an increased JPE in lead V1 with increasing numbers of BrS-associated risk alleles. Carrying five or six BrS-associated risk alleles increased the J-point in V1 with 17.1 μV (95% CI: 11.0–23.2, *P* < 0.001) (Table 4, Figure 2). The same additive effect was, however, not observed in lead V2 (see Supplementary Figure 3). Figure 3 shows that the number of risk alleles affected JPE in both genders.

**Table 4:** Additive effect of BrS-associated risk alleles on J-point elevation in lead V1.

**Number of alleles**	**N**	**Mean J-point elevation in lead V1, μV**	**95% CI**	***P*-value**
0	428	3.5	(0.7–6.3)	
1–2	3,575	7.4	(6.4–8.3)	0.0063^†^
3–4	1,912	8.3	(7.0–9.6)	<0.001^†^
5–6	69	17.1	(11.0–23.2)	<0.001^†^

*CI, confidence interval; N, total number of individuals. Variants: SCN5A rs11708996; G/C, risk allele C, SCN10A rs6800541; C/T, risk allele C, and HEY2 rs9388451, T/C, risk allele C*.

† *Statistically significant compared to zero alleles with a P-value < 0.05 with Dunnett's Post Hoc Test. Age and sex adjusted*.

### Effect on the PR-interval, QRS duration, and QTc interval

Homozygote carriers of the rs11708996 risk variant located intronic to *SCN5A* had a shortened QTc interval (*P* = 0.003). Both the rs11708996 and rs6800541 risk variants were associated with a prolonging effect on the PR interval (*P* < 0.001 and *P* < 0.001, respectively) and a prolonging effect on the QRS interval (*P* = 0.001 and *P* < 0.001, respectively). Carriers of the rs9388451 risk variant located downstream to *HEY2* had a prolonged PR interval (*P* = 0.03) and a shortened QRS interval compared with non-carriers (*P* = 0.02).

### UK biobank

**Figure 4:**
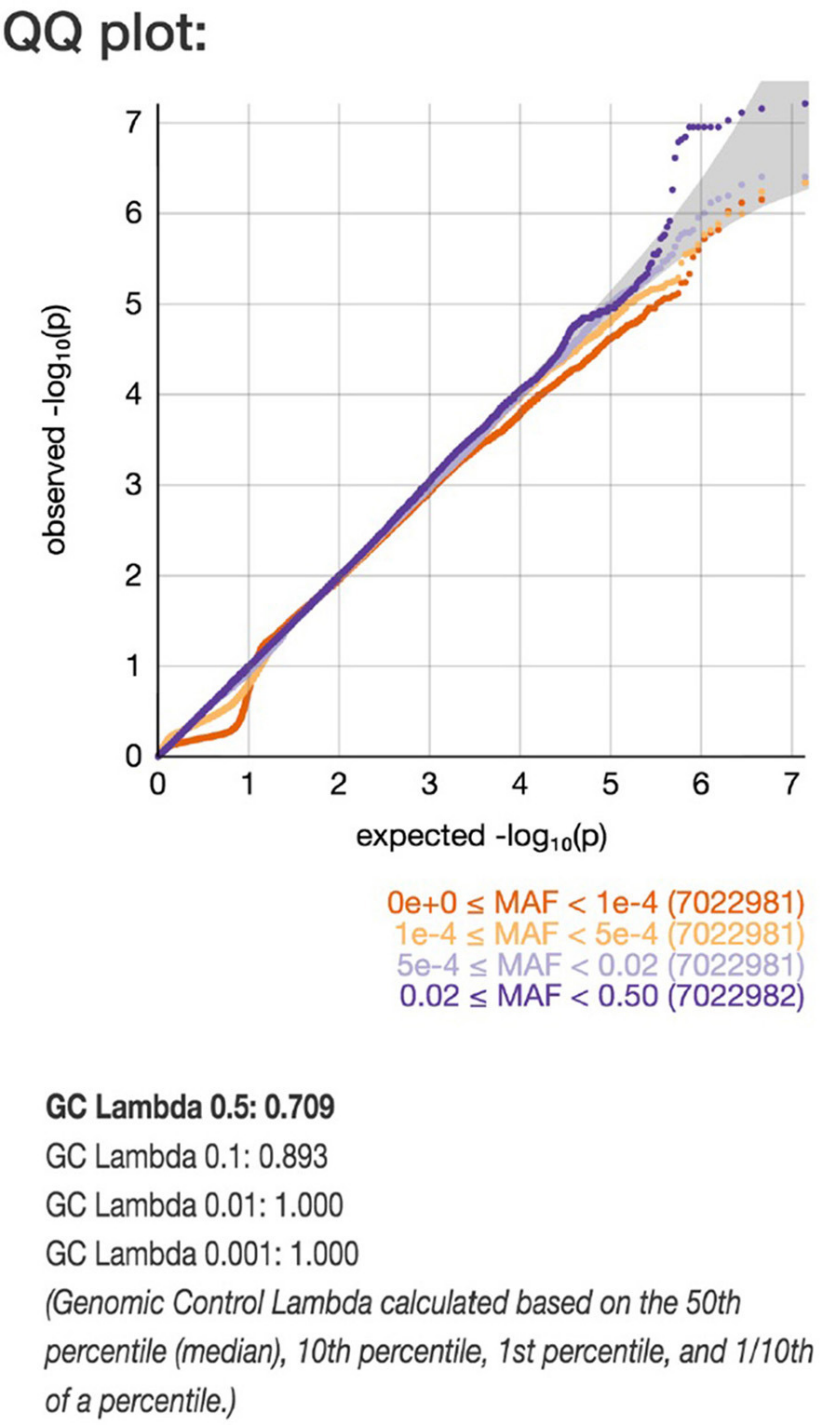
QQ plot of genome-wide single-variant association analyses in the UK Biobank cohort. Markers are stratified by MAF between 0.5–0.02 and 5 × 10^−4^ −1 × 10^−4^ shown with light blue and dark blue. Dots indicate observed *P*-values (–log10[*P*-value]) compared with those expected by chance. The gray line indicates the identity (no association) including the corresponding 95% CI under the null hypothesis (no association).

Using the UK Biobank data, analyzed with the Michigan PheWeb browser, we show that the rs9388451 genetic locus close to the *HEY2* gene is associated with cardiac arrest/VF ICD-9 code 427.4; 1,137 cases versus 380,919 controls, allele frequency 0.52, beta-value 0.12 corresponding to OR = 1.13 (95% CI: 1.08–1.18), *P* = 0.006). QQ plot and corresponding lambda values of a GWAS on ICD-9 code 427.4 are shown in Figure 4. A list of found associations with a *P*-value above 1.0 × 10^−5^ are shown in Supplementary Table 1.

## Discussion

In a large cohort representing the general middle-aged population in Denmark we found that carrying BrS-associated risk alleles in *SCN5A, SCN10A*, and *HEY2*, did not associate with an increased risk of death from all causes. Neither was there an increased risk regarding syncope or AF. Interestingly, we found that carrying an increasing number of BrS-associated risk alleles increased the JPE in lead V1 in an additive manner. Furthermore, the *HEY2* risk allele was associated with cardiac arrest/VF in a similar general population of the United Kingdom, indicating a potential lethal effect of the common genetic variant.

### J-point elevation and ST-T wave

Several studies have shown JPE to associate with an increased risk of SCD in the general population (Haïssaguerre et al., 2008; Tikkanen et al., 2009; Olson et al., 2011). We find three SNPs to have a modest effect on the JPE in the general population, indicating that there might be common variants associated with JPE in the general population. Interestingly, the effect of risk allele number on JPE was similar in both genders suggesting that the male dominance of BrS is unlikely to be mediated by either *SCN5A, HEY2*, nor *SCN10A*. Another SNP (rs6801957), which is in perfect linkage disequilibrium (LD) (*r*^2^ = 1.00) with rs6800541 intronic to *SCN10A*, has recently been associated with ST-T wave amplitudes in a large GWAS on European individuals, an ECG parameter closely related to the JPE (Verweij et al., 2016).

### PR, QRS, and QTc interval prolongation

We found a statistically significant prolongation of the PR interval when carrying the rs11708996 and rs6800541 risk variants located intronic to *SCN5A* and *SCN10A*, respectively. These findings are in line with the data presented by Pfeufer et al. (2010), where the authors found the *SCN5A* and *SCN10A* loci to prolong the PR interval and to decrease the risk of AF (Pfeufer et al., 2010). These findings have later been replicated in a Scandinavian cohort of lone AF patients (Andreasen et al., 2014). In agreement with our data, Sotoodehnia et al. (2010) have also showed the *SCN5A* and *SCN10A* variants, or a variant in high linkage, to prolong the QRS interval. A PR and QRS prolonging effect of BrS risk variants supports that the cardiac depolarization process has an important role in the BrS pathogenesis (Wilde et al., 2010).

A previous GWAS has associated the rs11129795 loci in *SCN5A* with a shortened QTc interval, in line with our results, however, the QT interval GWAS loci is not in LD with our *SCN5A* risk variant. The impact of the SNPs therefore serves to validate prior work.

The RAFs in our Danish study population resembles the RAFs of the control population in the original BrS GWAS (rs11708996; 0.15 vs. 0.15, rs6800541; 0.38 vs. 0.41, and rs9388451; 0.49 vs. 0.50), indicating that the populations are comparable.

### The role of BrS risk alleles in overall mortality

We found none of the three BrS risk alleles to have an effect on the risk of syncope, AF, or overall mortality in a general population of Denmark. In order to investigate a similar general population, we used 382,000 white British participants from UK Biobank, and found the *HEY2* risk allele rs9388451 to associate with cardiac arrest/VF, indicating a potential lethal effect of the common genetic variant. The *HEY2* risk allele has been analyzed in the GEVAMI study on Danes with ST-elevation myocardial infarction (STEMI) and has been associated with a borderline significant increased risk of VF [OR = 1.50 (95% CI: 0.96–2.40), *P* = 0.070 in 257 patients with VF caused by STEMI compared with 537 STEMI controls without VF; (Jabbari et al., 2017)], further supporting our findings.

As the average age at the end of follow-up in our study was almost 60 years, we could suspect potential BrS symptoms, due to the three BrS risk alleles alone, to have developed, bearing in mind that most BrS patients develop symptoms in their fourth decade of life (Hedley et al., 2009). However these SNPs had a significant effect on the JPE even in the general population, which may explain why they act as modifiers in BrS patients (Bezzina et al., 2013). This although common variants in general are thought to have small effect sizes. A recent study has shown rare variants previously associated with BrS to have no effect on syncope, malignant cardiac arrhythmia, and all-cause mortality in the general population (Ghouse et al., 2016). Diverging results in previous studies calls for studies on larger patient populations in order to understand the pathophysiological mechanisms of BrS.

### Limitations

In the Danish dataset only patients admitted to a hospital in relation to syncope was included in the analyses. Also, it is not possible with the current ICD-10 system to differentiate between syncope caused by arrhythmia and vasovagal episodes. Registry data on syncope was only available from the year 1994. The youngest patients were included from the age of 29 years and adverse life events might have occurred prior to the time of inclusion, leaving a potential survival bias.

The association of the *HEY2* loci with cardiac arrest/VF is obviously very interesting but the association needs to be replicated when an independent dataset of similar size becomes available.

## Conclusions

The three SNPs in *HEY2*, rs9388451, *SCN5A*, rs11708996, and *SCN10A*, rs6800541 are associated with significant electrocardiographic changes in ST elevation, PR interval, and QRS duration which may explain the effect in BrS patients. An association of the *HEY2* risk allele was found with cardiac arrest/VF in the larger UK Biobank population indicating a possible pathological role in cardiac arrhythmia.

## Data availability

Sequence data are made available at the European Genome-phenome Archive (EGA), which is hosted by the EBI and the CRG, under accession number EGAS00001003059.

## Ethics statement

The Inter99 study was approved by The Copenhagen County Ethical Committee (KA 98155) and the National Board of Health of Denmark. All participants have given their informed consent.

## Author contributions

MO, JK, and LA designed the study. JK coordinated and carried out the statistical analyses. CH, AL, OP, and TH provided the Inter99 population. SH and JS obtained funding. LA, JG, MS, GA, PR, JK, and MO drafted the manuscript. All coauthors critically revised the manuscript for intellectual content. MO and JK led the study together.

### Conflict of interest statement

The authors declare that the research was conducted in the absence of any commercial or financial relationships that could be construed as a potential conflict of interest.

## Footnotes

^1^*Genome-wide genetic data on* ~*500,000 UK Biobank participants* | bioRxiv [cited 2018 Jan 5];Available online at: https://www.biorxiv.org/content/early/2017/07/20/166298.

^2^*Efficiently Controlling for Case-Control Imbalance and Sample Relatedness in Large-Scale Genetic Association Studies* | bioRxiv [cited 2018 Jan 5];Available online at: https://www.biorxiv.org/content/early/2017/11/24/212357.
