# Supplementary material for: Brugada Syndrome-Associated Genetic Loci Are Associated With J-Point Elevation and an Increased Risk of Cardiac Arrest
Source: Front Physiol. 2018 Jul 10;9:894. doi: 10.3389/fphys.2018.00894 (PMC6048413; doi:10.3389/fphys.2018.00894)
Supplement: Supplementary file 1 [file Data_Sheet_1.docx]

**SUPPLEMENTARY MATERIAL**

**Supplementary Figures**

**Supplementary Figure 1**

**
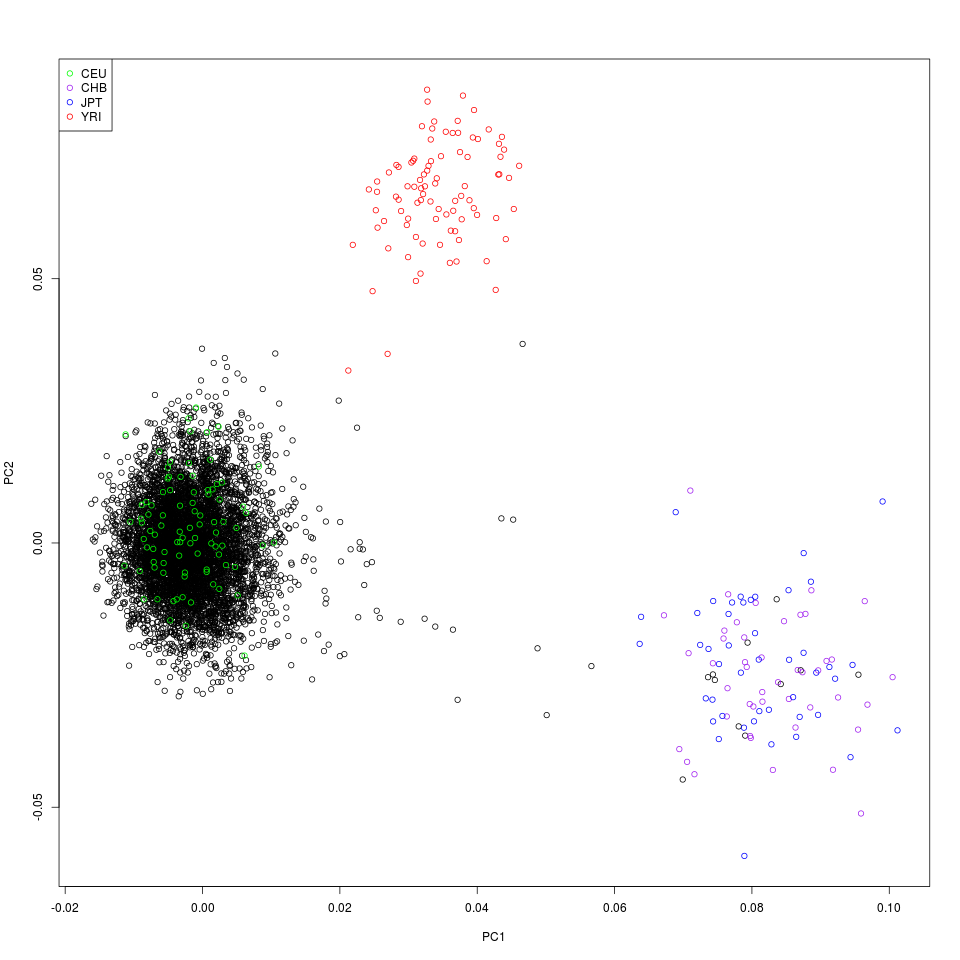
**

**Supplementary Figure 2**

**
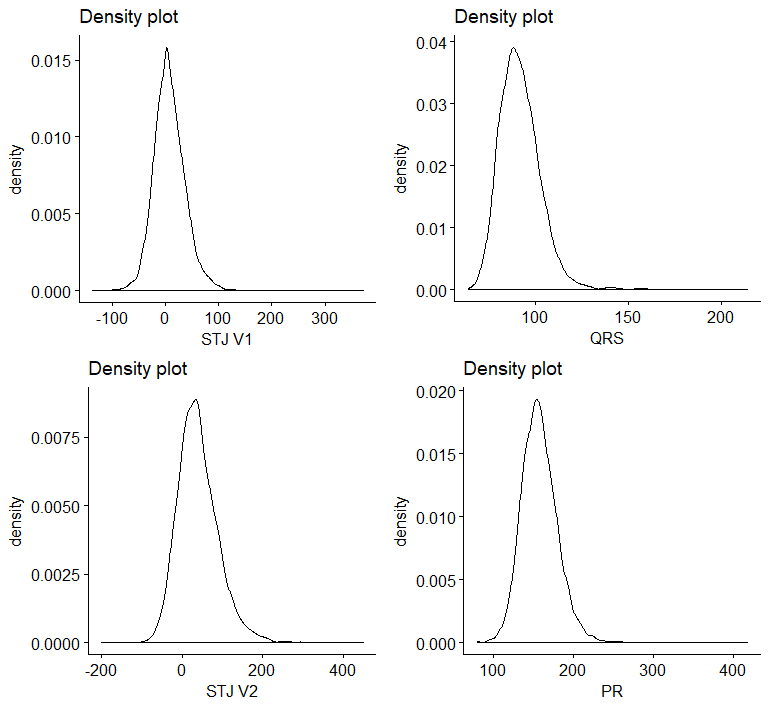
**

**Supplementary Figure 3**

**

**

**Figure legends**

**Supplementary Figure 1.** Principal component analysis (PCA) on Danish study cohort. Black circles represent study population, showing a considerable clustering with CEU.

CEU, Utah residents of northern and western European ancestry; CHB, Unrelated Han Chinese, from Beijing, China; JPT, Unrelated Japanese individuals from Tokyo, Japan; YRI, Individuals from Yoruba in Ibadan, Nigeria.

**Supplementary Figure 2.** Distribution plots of JPE in V1 and V2, PR-interval, and QRS-duration.

JPE, J-point elevation.

**Supplementary Figure 3.** The additive effect of carrying multiple BrS-associated risk alleles on the J-point elevation in lead V2. JPE (mean ± SE) as a function of number of risk alleles (*SNC5A* rs11708996, *SCN10A* rs6800541, *HEY2* rs9388451).

JPE, J-point elevation; SNP, single nucleotide polymorphism.

**Supplemental Tables**

**Supplementary Table 1**

| **Variant** | **Nearest Gene(s)** | **MAF** | **P-value** |
| --- | --- | --- | --- |
| 5:23.707.356 A / G (rs4604157) | *PRDM9* | 00.46 | 6.1e-8 |
| 8:136.784.310 G / A (rs186328106) | *KHDRBS3* | 0.021 | 2.4e-7 |
| 6:99.533.932 G / A (rs140213193) | *FBXL4* | 0,056944444 | 3.9e-7 |
| 4:94.686.652 G / A (rs148365102) | *GRID2* | 0,050694444 | 3.9e-7 |
| 16:78.342.694 C / T (rs189352573) | *WWOX* | 0.000055 | 4.5e-7 |
| 7:107.702.689 A / T (rs538348008) | *LAMB4* | 0.00036 | 4.5e-7 |
| 4:9.184.405 T / C (rs3961218) | *FAM90A26* | 00.44 | 5.4e-7 |
| 10:15.839.054 A / G (rs571391502) | *FAM188A* | 0.00032 | 5.7e-7 |
| 3:136.586.707 G / A (rs112515324) | *NCK1* | 0,066666667 | 6.3e-7 |
| 8:130.549.386 G / C (rs557707067) | *GSDMC* | 00.33 | 7.6e-7 |
| 1:114.020.708 T / G (rs540824367) | *MAGI3* | 0.00011 | 7.6e-7 |
| 18:6.567.219 G / A (rs4503892) | *L3MBTL4* | 0.000054 | 9.4e-7 |
| 14:36.724.222 G / A (rs145083903) | *MBIP* | 00.17 | 9.8e-7 |
| 2:201.175.650 T / C (rs768813915) | *SPATS2L* | 0.00046 | 1.0e-6 |
| 18:56.936.744 G / C (rs200098438) | *RAX* | 00.11 | 1.1e-6 |
| 1:215.828.004 T / C | *USH2A* | 0.00013 | 1.3e-6 |
| 1:241.995.569 A / T (rs188036036) | *EXO1* | 0.00041 | 1.5e-6 |
| 1:167.625.478 C / T (rs571013158) | *RCSD1* | 0.000034 | 1.5e-6 |
| 1:105.794.483 C / T (rs115398440) | *AMY1C* | 00.12 | 1.5e-6 |
| 22:39.360.080 T / C (rs147522059) | *APOBEC3A* | 0.000059 | 1.6e-6 |
| 12:84.715.526 A / G (rs191847406) | *SLC6A15* | 00.19 | 1.6e-6 |
| 3:186.762.665 C / G (rs761431492) | *ST6GAL1* | 0.00066 | 1.6e-6 |
| 18:8.588.315 G / A (rs571004558) | *RAB12* | 0.00044 | 1.7e-6 |
| 5:145.279.898 C / T (rs111544350) | *GRXCR2* | 00.24 | 1.7e-6 |
| 5:12.050.910 T / C (rs7720978) | *CTNND2* | 00.20 | 1.7e-6 |
| 4:138.428.490 C / T (rs78905079) | *PCDH18* | 0.033 | 1.8e-6 |
| 9:30.459.302 T / G (rs113768591) | *LINGO2* | 00.43 | 1.9e-6 |
| 1:212.637.895 C / A (rs564436560) | *NENF* | 0.000079 | 1.9e-6 |
| 1:176.337.093 T / C (rs557570462) | *PAPPA2* | 0.00027 | 2.2e-6 |
| 3:135.739.897 A / G (rs189056462) | *PPP2R3A* | 0,044444444 | 2.3e-6 |
| 4:30.279.879 G / A (rs79740630) | *PCDH7* | 0.000028 | 2.5e-6 |
| 20:18.463.897 C / T (rs2424212) | *POLR3F* | 00.11 | 2.6e-6 |
| 7:137.929.787 A / G (rs751047476) | *AKR1D1* | 0.00012 | 2.6e-6 |
| 15:29.269.922 G / A | *APBA2* | 0.00042 | 2.7e-6 |
| 7:79.624.842 T / A (rs570836132) | *GNAI1* | 0.00014 | 2.8e-6 |
| 3:88.919.050 G / C (rs919483029) | *EPHA3* | 0.00091 | 2.8e-6 |
| 1:87.409.121 C / T (rs547078464) | *HS2ST1* | 0.000097 | 3.0e-6 |
| 16:57.660.088 C / T (rs572846812) | *ADGRG1* | 0,050694444 | 3.2e-6 |
| 18:5.381.913 T / A (rs183219815) | *EPB41L3* | 00.54 | 3.4e-6 |
| 11:10.561.551 T / C (rs535266414) | *RNF141* | 0.00053 | 3.4e-6 |
| 2:239.742.054 C / T (rs532111934) | *TWIST2* | 0.00016 | 3.5e-6 |
| 16:61.548.350 A / C (rs181012867) | *CDH8* | 0.00055 | 4.0e-6 |
| 3:50.558.373 G / A (rs79402695) | *CACNA2D2* | 0.019 | 4.0e-6 |
| 1:238.056.130 C / T (rs75524044) | *ZP4* | 00.59 | 4.0e-6 |
| 12:81.393.824 G / A (rs187043090) | *ACSS3* | 00.14 | 4.2e-6 |
| 10:101.921.224 A / G (rs533477921) | *ERLIN1* | 0.00057 | 4.2e-6 |
| 1:196.259.038 G / C (rs142530070) | *KCNT2* | 00.50 | 4.4e-6 |
| 2:235.714.195 G / A (rs151302062) | *SH3BP4* | 0,054861111 | 4.5e-6 |
| 6:92.432.415 C / T (rs139027335) | *MAP3K7* | 0.000033 | 4.6e-6 |
| 5:64.592.319 A / C (rs6449780) | *ADAMTS6* | 0.050 | 4.6e-6 |
| 4:21.945.400 G / A (rs187327991) | *KCNIP4* | 00.31 | 4.8e-6 |
| 8:146.228.779 T / A (rs77308683) | *C8orf33* | 0.076 | 4.9e-6 |
| 10:65.770.182 A / C (rs186615124) | *REEP3* | 0.00061 | 5.0e-6 |
| 18:69.679.792 A / G (rs9962621) | *CBLN2* | 0.00019 | 5.1e-6 |
| 12:83.923.896 A / G (rs534712875) | *TMTC2* | 00.15 | 5.3e-6 |
| 16:31.588.797 G / A (rs116921468) | *AHSP* | 0.00035 | 5.5e-6 |
| 9:126.081.452 G / A (rs79389622) | *CRB2* | 0.00091 | 5.5e-6 |
| 7:96.078.410 C / T (rs776563573) | *SHFM1* | 00.12 | 5.5e-6 |
| 2:74.071.111 A / G (rs766927582) | *STAMBP* | 0.00057 | 5.5e-6 |
| 16:82.820.768 C / G (rs35518553) | *CDH13* | 00.14 | 5.6e-6 |
| 12:56.593.502 C / T (rs766758157) | *RNF41* | 0.00035 | 5.6e-6 |
| 9:11.976.057 A / G (rs542850458) | *TYRP1* | 0.000087 | 5.7e-6 |
| 10:105.310.864 C / A (rs533990469) | *NEURL1* | 0.00042 | 5.8e-6 |
| 7:126.956.446 C / T (rs544869464) | *ZNF800* | 0.00033 | 5.8e-6 |
| 7:33.448.445 G / T (rs77929072) | *BBS9* | 0.00010 | 5.8e-6 |
| 6:4.026.486 C / T (rs557767583) | *PRPF4B* | 00.14 | 6.1e-6 |
| 13:81.597.179 A / T (rs9601519) | *SPRY2* | 0.013 | 6.2e-6 |
| 6:134.542.302 C / T (rs761408742) | *SGK1* | 0.00036 | 6.2e-6 |
| 17:48.080.099 A / G (rs75613387) | *DLX3* | 00.11 | 6.3e-6 |
| 5:12.746.465 G / C (rs535627937) | *CTNND2* | 0.00051 | 6.4e-6 |
| 1:115.188.452 C / A (rs577402946) | *DENND2C* | 00.44 | 6.4e-6 |
| 16:9.140.252 C / T (rs368782031) | *C16orf72* | 0.00021 | 6.5e-6 |
| 4:148.931.201 T / C (rs751606099) | *ARHGAP10* | 0.00016 | 6.5e-6 |
| 10:31.105.495 C / T (rs139801147) | *ZNF438* | 0.00015 | 6.6e-6 |
| 17:47.147.738 C / T (rs768660392) | *IGF2BP1* | 0.00012 | 6.7e-6 |
| 7:26.001.671 C / T (rs183533270) | *NFE2L3* | 0,058333333 | 6.7e-6 |
| 2:6.053.940 G / A (rs772624709) | *SOX11* | 0.00018 | 7.0e-6 |
| 17:73.819.159 G / A (rs146545692) | *UNK* | 0.012 | 7.1e-6 |
| 5:146.114.488 C / T | *PPP2R2B* | 0.00020 | 7.2e-6 |
| 5:81.158.986 C / T (rs112894818) | *ATG10* | 0.012 | 7.2e-6 |
| 4:92.946.230 C / T (rs115862313) | *GRID2* | 0,057638889 | 7.4e-6 |
| 4:13.222.532 G / A (rs148130900) | *RAB28* | 0.00012 | 7.4e-6 |
| 5:68.425.818 T / A (rs879454483) | *SLC30A5* | 0.00066 | 7.5e-6 |
| 4:137.530.944 G / C (rs560813464) | *PCDH18* | 0.00028 | 7.5e-6 |
| 11:108.100.868 C / T (rs140198723) | *ATM* | 0.00032 | 7.6e-6 |
| 11:88.888.124 T / C (rs374597961) | *TYR* | 0.000039 | 7.6e-6 |
| 12:109.925.439 T / G (rs78113238) | *UBE3B* | 0,061111111 | 7.7e-6 |
| 20:53.851.865 G / T (rs558232723) | *DOK5* | 00.12 | 7.9e-6 |
| 8:20.364.618 G / A (rs181549727) | *LZTS1* | 0.000031 | 8.0e-6 |
| 13:100.468.733 G / A (rs780832822) | *CLYBL* | 0.00015 | 8.1e-6 |
| 3:74.888.177 C / T (rs149143311) | *CNTN3* | 0.000041 | 8.1e-6 |
| 1:89.408.567 C / T (rs371283861) | *KYAT3* | 0.00089 | 8.1e-6 |
| 15:89.645.230 A / G (rs293380) | *ABHD2* | 00.46 | 8.4e-6 |
| 2:170.053.801 C / T (rs78745237) | *LRP2* | 0.00036 | 8.4e-6 |
| 2:67.799.461 G / C (rs565459307) | *ETAA1* | 00.15 | 8.4e-6 |
| 12:82.009.887 G / C (rs192283973) | *PPFIA2* | 00.12 | 8.5e-6 |
| 5:324.926 G / A (rs577752017) | *AHRR, PDCD6* | 0.000038 | 8.5e-6 |
| 9:74.369.850 T / C (rs770529239) | *TMEM2* | 0.000088 | 8.6e-6 |
| 6:78.032.592 A / T (rs185997591) | *HTR1B* | 00.23 | 8.7e-6 |
| 2:8.166.784 A / G (rs116576077) | *ID2* | 0.000036 | 8.7e-6 |
| 6:45.927.893 A / G (rs553717786) | *CLIC5* | 0.00024 | 8.8e-6 |
| 15:40.543.563 G / A (rs149280298) | *BUB1B-PAK6, PAK6* | 0.000063 | 8.9e-6 |
| 4:95.306.168 A / C (rs142912614) | *HPGDS* | 0.012 | 8.9e-6 |
| 11:39.213.808 C / T (rs10837122) | *LRRC4C* | 00.49 | 9.0e-6 |
| 1:173.647.242 C / A (rs184343657) | *ANKRD45* | 00.44 | 9.2e-6 |
| 20:61.150.998 G / A (rs781794954) | *GATA5* | 0.00025 | 9.4e-6 |
| 10:4.846.183 G / T (rs565035936) | *AKR1E2* | 0.000094 | 9.4e-6 |
| 1:63.461.726 T / C | *ATG4C* | 0.000087 | 9.5e-6 |
| 16:81.919.149 C / G (rs150486474) | *PLCG2* | 00.19 | 9.6e-6 |
| 11:123.018.876 C / T (rs764648920) | *CLMP* | 0.00028 | 9.6e-6 |
| 3:10.301.768 T / C (rs375232465) | *RP11-438J1.1, TATDN2* | 0.00047 | 9.6e-6 |
| 1:246.005.947 G / A (rs192402361) | *SMYD3* | 0.00038 | 9.6e-6 |
| 3:73.350.940 G / C (rs182608200) | *PDZRN3* | 0.00012 | 9.7e-6 |
| 14:73.431.596 C / T (rs532992562) | *ZFYVE1* | 0.00069 | 9.9e-6 |
| 6:128.198.785 C / T (rs773719115) | *THEMIS* | 0.000084 | 9.9e-6 |
| 3:41.128.725 C / T (rs148954094) | *CTNNB1* | 0,04375 | 9.9e-6 |

MAF; minor allele frequency.
